# The Persistence of Memory: Behavioral Analysis and Arm Usage of a Nine-Armed *Octopus vulgaris*

**DOI:** 10.3390/ani15071034

**Published:** 2025-04-03

**Authors:** Sam Ellington Soule, Miguel Cabanellas-Reboredo, Ángel F. González, Hidde Juijn, Jorge Hernández-Urcera

**Affiliations:** 1 ECOBIOMAR Research Group, Institute of Marine Research (IIM-CSIC), Eduardo Cabello 6, 36208 Vigo, Spain; afg@iim.csic.es; 2 Centro Oceanográfico de Illes Balears (COB-IEO), CSIC, Moll de Ponent s/n, 07015 Palma de Mallorca, Spain; miguel.cabanellas@ieo.csic.es (M.C.-R.); hiddejuijn@gmail.com (H.J.)

**Keywords:** cephalopods, *Octopus vulgaris*, nine-armed *Octopus*, bifurcation, arm use, behavior, adaptability, pain-associated memory

## Abstract

Anatomical abnormalities in octopuses, whose behavior is facilitated by flexible, neuron-rich arms, offer insights into life histories and the neurological implications of understudied conditions such as bifurcation. Although documentation is scarce, here we present in situ videos of nine-armed *O. vulgaris* with a functional bifurcated R1 arm. Analysis using RDAs and GLMs investigated the impact of the bifurcated arm on behavior and examined changes during growth. Analysis revealed a differential usage of between the bifurcated arms in addition to an initial specialization of the bifurcated arms for actions below the body, decreasing over time for only one of the arms as grew. Further, bifurcated and regrown arms were utilized more in safe behaviors than risky ones, with more severely injured arms showing a higher frequency of use in safe behaviors. These findings contribute to the growing knowledge of arm usage in octopuses, suggesting that arm bifurcation may lead to branchial neural differentiation and potentially indicate post-traumatic associated in *O. vulgaris*.

## Simple Summary

Octopuses have flexible arms with many nerve cells, allowing them to explore and interact with their environment in unique ways. Sometimes, these animals develop unusual features, such as extra or split arms, but little is known about how this affects their movement and behavior. This study examined a wild *Octopus* with a naturally split arm, using underwater video recordings to observe how it used its arms over time. The results showed that the split arms were initially used more for actions beneath the body but became less specialized as the octopus grew. Additionally, both split and regenerated arms were used more often in safe situations rather than risky ones, especially when the arms had been previously injured. These findings suggest that octopuses may adapt their arm use based on injury and recovery, possibly showing changes in how their nervous system controls movement. Understanding these adaptations can provide insight into how animals respond to physical challenges and could even inspire new designs in robotics and prosthetics by mimicking the recovery abilities of octopus arms.

## 1. Introduction

The molluscan class Cephalopoda, and particularly individuals within the order Octopoda, have been celebrated for their complex body patterning and unique morphology, including their regeneratable arms. Despite considerable research on the regeneration process, the specific molecular pathways and morphological modifications involved remain unclear [1,2,3]. Occasionally, during regeneration, octopuses’ arms, particularly their tips, undergo splitting and branching in their growth, causing conditions such as bifurcation, wherein the arm branches once, producing two arms in place of one. Although it is still uncertain, the molecular mechanism for this abnormal growth has been hypothesized as being due to a mutation in the hox group of the homeobox genes [4,5]. Homeobox genes direct the formation of many body structures in early development, while the expression of the hox group provides the basis for the anterior versus posterior axis specification [6]. In invertebrates, the mutation of these genes can lead to the growth of an extra and typically non-functional body part [7]. An alternative molecular pathway associated with abnormal growth in Cephalopods is the Hedgehog (Hh) signaling pathway [8]. Present in both embryonic and adult tissues, it similarly mediates processes such as development, morphogenesis, and growth [9]. This abnormal growth was noted during the transplantation of tissue expressing the Hh gene family onto the posterior side of a stage 17 limb bud in *Sepia officinalis*, which, within 7 of the 14 trials, resulted in a posteriorly duplicated limb [8]. Despite the identification of these mechanisms, as so few individuals with arm abnormalities have been recorded, with even fewer found alive, very little is still known about this process. Nevertheless, as the arms of an octopus help contribute to their incredible behavioral flexibility, the addition of an extra appendage has been speculated as altering the complex suite of behaviors [10].

Although the study of this behavioral suite is comprehensive, recent reviews have pointed to a need to study individuals in situ or within the field, as current methods use ex situ or laboratory experiments to examine behavior [11,12]. These approaches cause concerns in providing environmental context to observed behaviors. To help fill the gaps in environmentally relevant studies and to describe and analyze this rare abnormality, this study presents videos of a living cephalopod with a fully functional bifurcated arm in the wild. This paper aims to employ behavioral quantification methods to describe and examine the behavioral repertoire of this unique individual via videos collected by citizen science. Special attention was directed towards behaviors and actions involving arm usage, and a particular emphasis was placed on the two bifurcated arms. The research questions investigated are as follows: (1) Does the presence of a fully functional bifurcated arm alter the behavior of *Octopus vulgaris*? (2) To what extent is the bifurcated arm used in different behaviors? (3) Does the usage of the arms change over time?

## 2. Materials and Methods

### 2.1. Study’s Subject

**Figure 1:**
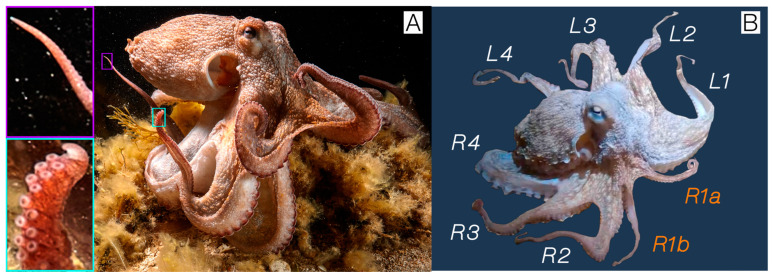
(**A**) Photo taken on 2 May 2022, showing the hectocotylized R3 arm (blue) and a normal arm tip (purple). (**B**) Photo from 28 January 2022, displaying terminology used for arms, with bifurcated R1 arms in orange.

All videos in this study are of the same male *O. vulgaris*. Its gender was determined based on photo analysis showing a hectocotylized R3 arm (Figure 1A). Although its cause was not captured on video, this individual was presumed to have lost arms R1, R2, R3, and tips of R4 and L1 in a potential past predator encounter. During regrowth, arm R1 bifurcated, producing arms R1a and R1b, while all other arms regrew normally (Figure 1B). The individual was recorded during ~2 h dusk dives wherein the subject was slowly approached at substrate level and followed from 0.5 m away to not disturb or affect the observed behaviors.

### 2.2. Study Site

All the data used in the current study were collected within Ibiza, Spain (39°00′36″ N 1°17′54″ E). The site is an inlet cove with a depth ranging from 0 to 25 m, containing mixed substrate and sand at shallower depths and *Posidonia oceanica* meadows and larger rock formations deeper. At this site, suitable habitat and low fishing pressure allow the inhabitation of various species of cephalopods (*Callistoctopus macropus*, *Loligo vulgaris*, *Sepiolda rondeletii*) and other marine species (*Sphyraena sphyraena*, *Muraena helena*, *Conger conger*). The filming area spanned approximately 2083.8 m^2^, wherein 6 dens, all shallower than 5 m, were identified for this individual throughout the period.

### 2.3. Quantification of Behavioral Data

Using the open-source software *Behavioural Observation Interactive Research Software* (BORIS v. 8.16.6) [13], the occurrence and duration of behaviors were analyzed from >10 h of video records (23 videos). To do this, an ethogram created using behaviors previously noted in various *Octopus* species (Table S2) was inputted into the software, and specific keys were associated with each of the actions and behaviors in the ethogram. This allowed each behavior to be recorded as a singular event (from the beginning to the finish of a concrete behavior) within the entire observation of each video. Events were classified into two types based on their association with duration: point events (without durations) and state events (with durations). In this sense, inside each of the 24 videos, the events were identified, and the usage/non-usage of each of the individual’s 9 arms were registered through a binomial response variable matrix. When new or altered behaviors not previously noted in the catalog were observed, they were later added to the ethogram for further consideration. Only video segments where a minimum of 3 arms were visible for the entire behavioral sequence were analyzed (see Videos S1 and S2 for examples of usable and unusable segments). Videos were analyzed at a maximum speed of 50 Frames Per Second (FPS), often frame by frame, to approximate arm movements accurately. Time budget data, exported as .csv files from BORIS, were used to create the final dataset.

### 2.4. Data Manipulation and Exploration

BORIS-generated outputs underwent data manipulation and exploration using R Studio version 2022.07.2. A binomial response variable matrix was crafted to signify arm usage (1) or non-usage (0) in identified events across the 23 videos. The video record dates were transformed into a continuous variable (days) for testing arm usage over time (with video 1: day 1 to video 24: 147 days). Behaviors were categorized into specific groups (Behavior Category) to address scientific questions about arm use (Table S1). Safe behaviors involved those wherein the arms were held closer to the body [14], while risky behaviors included actions with potential predator interactions and arm extensions away from the body.

After preliminary data inspection and visualization via triplots, noise or irrelevant behaviors were excluded based on three criteria: (1) relevance to arm use and scientific questions, (2) having more than 9 total events in all videos, and (3) completion in all videos. A new row for arm R1 was created by combining and correcting arms R1a and R1b for cohesive results, facilitating comparisons with past and future studies of arm usage in *O. vulgaris*, with or without bifurcated arms. In cases where behavioral categories showed no distinct trends for R1a versus R1b, R1 was used for analysis.

### 2.5. Preliminary Analysis and Arm Groupings

Arm usage and association within specific behaviors involving the scientific questions were analyzed and compared for each individual arm through the metrics of the total number of occurrences and the total duration of each arm within behavioral categories previously determined to address scientific questions. The metrics were later confirmed using a Redundancy Analysis (RDA) for all behaviors and the specific behavioral categories. From the general RDA on all behaviors, the even distribution of events associated with *Foraging*, *Exploration*, and *Locomotion* facilitated the combination of bifurcated arms into R1 during the specific RDAs for these categories. For *Under Web* actions and *Safe* vs. *Risky* behaviors, R1a and R1b were analyzed separately as these seemed to show differentiation in usage when an RDA was run with only bifurcated arms. When fitting Generalized Linear Models (GLMs), R1a and R1b were run separately for each behavioral category besides *Locomotion*, as there were no expected variations in usage between the two arms.

### 2.6. Statistical Analysis

Various Redundancy Analyses (RDAs; [15]) were used to visualize and test potential associations among the arms usage with behavior and behavioral categories. A preliminary RDA was run and visualized using a correlation triplot, which included all behaviors except those that did not meet the criteria previously explained. Following this initial step, additional behaviors were filtered out due to their high autocorrelation with behaviors more central to the research questions.

Before the RDA was run, all events were aggregated by behavior and time, which produced a table wherein total counts for behavior within each video were made. The RDA was completed using *vegan’s rda* function [16], with behaviors and behavior categories as explanatory variables, and the matrix of arm usage by event in each video as a response variable (transformed using the Hellinger-transform method; [15,17]). The potential variance conferred by time, as days after the first videos, was controlled by including it in the model as a condition factor.

The model was first assessed by the examination of the bimultivariate redundancy statistic—the amount of variance of the response matrix explained by the explanatory variables—equivalent to the R2 value in multiple regression [15]. However, the bimultivariate redundancy statistic of an RDA is biased like the ordinary R2 of multiple regression, and for the same reason [18]. This bias was fixed by adjusting the bimultivariate redundancy statistic using Ezekiel’s formula achieved through vegan’s function *RsquareAdj* [16,19]. The significant association of behaviors with arms usage was assessed using permutation tests implemented in the *anova* function. Lastly, the adequacy of the fitted RDA model was confirmed by assessing the linear relationships among explanatory variables using variance inflation factors (VIFs; [15]).

In addition, binomial regression via Generalized Linear Models (GLMs, fitted using *lme4* library in R software v. 2022.07.2 [20]) were used to test for potential changes in arm use over time and differences between probability of arm usage between the bifurcated arms. To conduct this, the binomial response of each arm (used/unused) was individually tested for specific behaviors or behavior categories in the function of time (from day 1 to day 147) that related to the scientific questions. Shapiro–Wilk tests were additionally used to assess the normality of the residuals. To visualize the data and test for differences between bifurcated arms, Logistic Regression Plots were used, which were then compared using a two-sample z-test for proportions.

## 3. Results

Videos were recorded from 13 December 2021 to 8 May 2022, a total of 147 days (including the final video, which was removed later due to unusable content). The distribution across the months was as follows: December (4.17%), January (16.67%), February (37.5%), March (25%), April (12.5%), and May (4.17%) (Table 1).

Of these videos, the total video duration ranged from 177 to 2686.10 s. As not all the content in each video was usable due to the arms not being visible, the percentage analyzed differed with minimums of 38.1% analyzed in video 18 and a maximum of 98.8% analyzed in videos 8 and 21 (mean: 88.3%). This led to 6642 events for analysis (Table 1).

Of these events, 38% were Postural Arm Components, 24.8% were Foraging behaviors, 20% were Exploration, 8% were Locomotion behaviors, 5.4% were Feeding, 0.8% were Arm Actions, 0.8% were Body Patterning, 0.3% were Alert behaviors, and 0.03% were Cleaning behaviors (see Table S1 for classification of Behavioral Categories). The behavioral categories mainly focused on in this study—Postural Arm Components, Foraging, Exploration, Locomotion, and Feeding—accounted for 96.1% of all the total events.

### 3.1. Arm Usage and Their Behavioral Associations

When examined within all recorded events, although increased usage of anterior arms over posterior arms was detected, R3 was favored in its occurrence of use over L2 (2472 vs. 2425 times used), albeit with a shorter duration (1423.7 vs. 1521.3 s) (Figure S1). On a sagittal plane, right-side arms were favored with both higher occurrences and durations of use (12,061 vs. 8051 times used, respectively). Finally, the arms with the highest usage were L1 and R1a (Figure S1).

**Figure 2:**
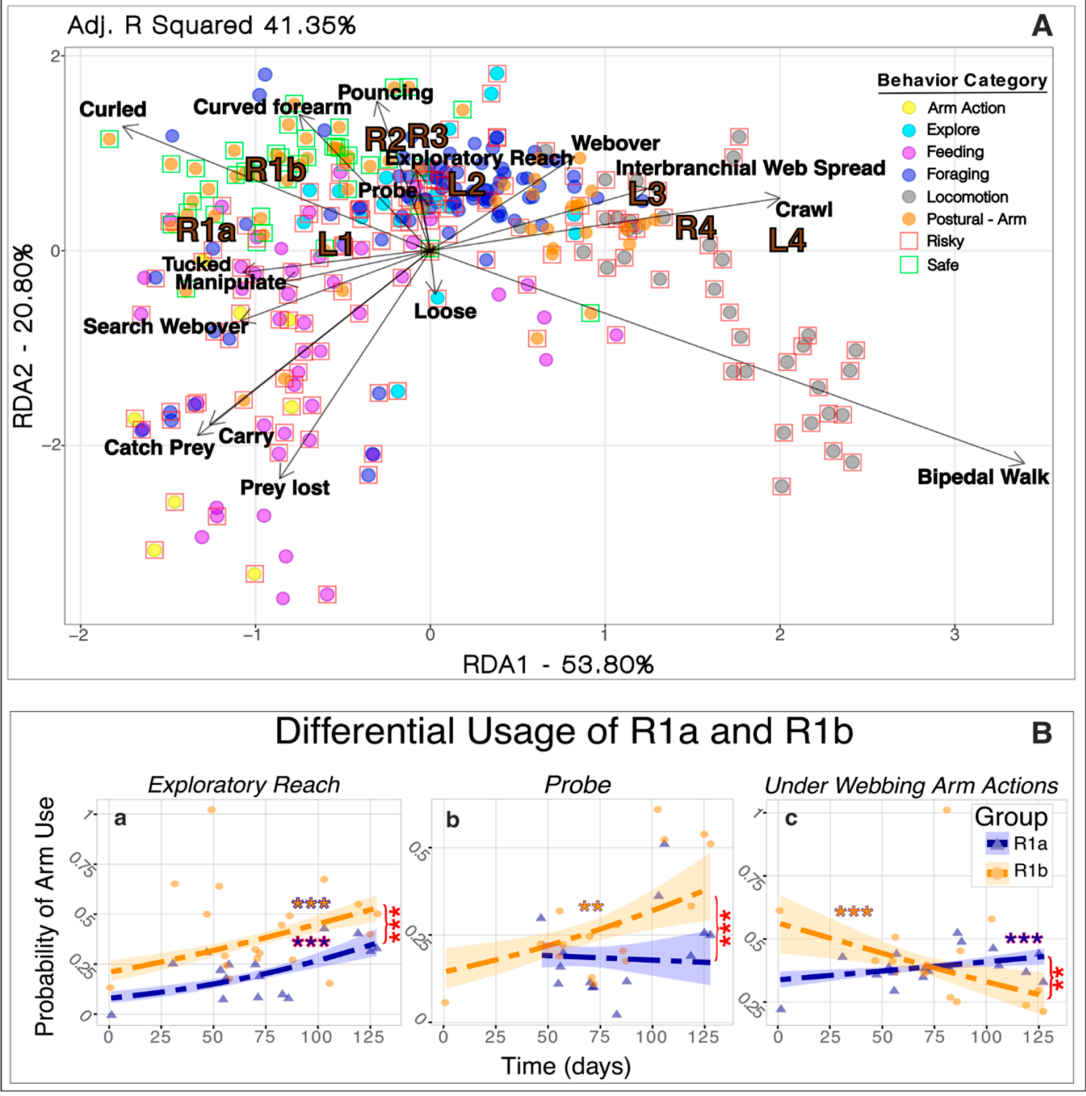
(**A**) Association of filtered behaviors with arms (R1a, R1b, R2, R3, L1, L2, L3, L4). Arrows indicate the direction and magnitude of behaviors associated with arms, while each point represents events aggregated by behavioral categories. Behavioral categories are denoted by colored circles, while risky and safe events are denoted by red and green boxes. (**B**) Differential usage of bifurcated arms over time (in days) with logistic regression model fit for selected behaviors (**a**–**c**). Orange and blue asterisks represent significant regression curves between respective arms and use over time, and the shaded areas indicate the confidence intervals of the model. Red asterisks represent significant differences between each arm’s probability of use within the respective behaviors. The significance level is represented by an asterisk as follows: ** *p* < 0.001, ***p < 0.0001.

Redundancy Analysis for behaviors related to the scientific questions significantly demonstrated associations of behaviors and their categories with arms (RDA, *p* < 0.001; Figure 2A). The RDA explained an adjusted 41.35% of the total variance of the arm usage response matrix. Of this percentage, 53.80% was explained by the first axis (RDA1), while 20.80% was explained by the second axis (RDA2), resulting in a total of 76% explained by the first two axes. *Locomotion* behaviors, particularly *Bipedal Walk* and *Crawl*, were more closely associated with the posterior arms, particularly L4 and R4. *Bipedal Walk* explained most of the variation along the first axis, suggesting its nearly exclusive use by the back two arms, while crawling was linked to other predominantly posterior arms. *Foraging* and *Exploratory* behaviors, namely, *Exploratory Reach*, *Probe*, and *Pouncing* were all associated with R2, L2, R3, L2, and to a lesser extent R1a and R1b; with R1b showing a significantly higher probability of use over time for *Exploratory Reach* and *Probe* than R1a (GLM, *p* < 0.001; Figure 2B(a,b)). Feeding behaviors and behaviors that occurred underneath the webbing, especially *Manipulate*, were associated with the bifurcated arms and L1, though favoring R1a and L1 to R1b with a significant difference between the probability of use overtime between R1a and R1b (GLM, *p* < 0.001; Figure 2B(c)). In the postural categories, *Arm(s) Curled* and *Arm(s) Curved* showed a heightened preference for using the bifurcated arms; however, its distribution throughout the graph is wide (Figure 2A).

### 3.2. Risky and Safe Usage

Analysis of usage revealed that right-sided arms displayed a cautious tendency, strongly associated with *Safe* behaviors, while left arms were linked to *Risky* behaviors. RDAs for the *Safe* behaviors showed a significant association for both right- and left-side arms (RDA, *p* < 0.001; Figure 2A). However, the adjusted bimultivariate redundancy statistic values (representing the amount of variance of the response matrix explained by the explanatory variables) differed, exhibiting a greater explanation of the model for the right-side arms (40%) than the left-side arms (18%). This association was further exemplified via the occurrence and duration of arms within *Safe* behaviors, with the right side showing greater values than the left for every arm except R4 (Figure S2A).

### 3.3. Bifurcated Arms’ Specialized Use

The association of *Under Web* actions with bifurcated arms was observed in the general RDA (Figure 2A). Analysis of actions involving arm movements under the webbing—*Search Webover*, *Manipulate*, and *Arm(s)-Tucked Under*—revealed a preference for anterior arms, except for arm R2. Arm R1a was the most frequently used (414 observations), followed by R1b (302 observations) and anterior left arms (Figure S2B). Arm L4 was the least utilized posterior arm (37 observations). The use of arm R1a in these behaviors significantly increased (GLM, *p* < 0.001; Figure 2B(c)) as the arm grew over time, while significantly decreasing over time for R1b (GLM, *p* < 0.001; Figure 2B(c)).

### 3.4. Behavioral Change over Time

**Figure 3:**
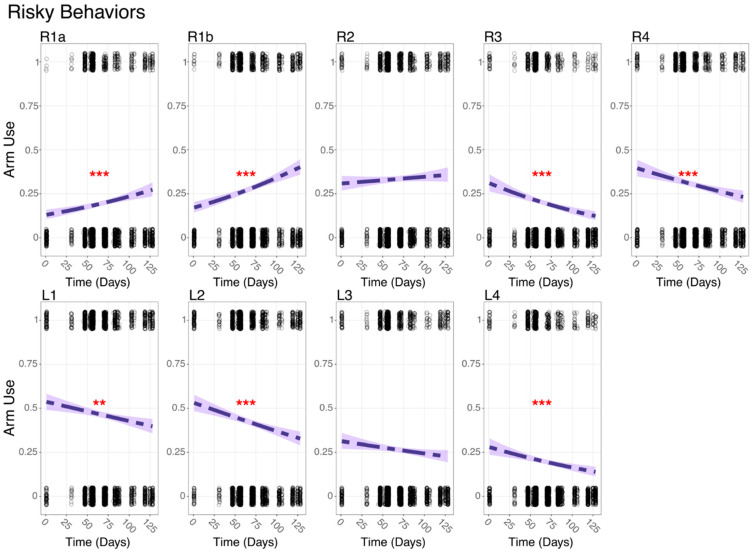
Summary plots of Risky Behavior usage over time for each arm. The logistic regression models were fitted to assess the change in behavior usage over time. Points represent observed data, and shaded areas represent the confidence intervals of the fitted models. The significance level is represented by an asterisk as follows: ** *p* ≤ 0.001, *** *p* ≤ 0.0001.

In *Risky* behaviors characterized by extended arm use or interactions with other species, R1a and R1b showed a significant increase in usage over time (GLM, *p* < 0.001; Figure 3), while R2 increased without statistical significance. All other arms, except L3, whose decrease was insignificant, exhibited a significant decrease in usage in *Risky Behaviors* over time (GLM, *p* < 0.001; Figure 3). In *Safe* behaviors, the anterior arms on the octopus’s right side (R1a, R1b, and R2) exhibited significant decreases in usage over time (GLM, *p* < 0.0001; Figure S3), while the posterior arms showed no significant change. On the left side, only L4 demonstrated a significant increase over time (GLM, *p* < 0.001; Figure S3).

### 3.5. Behavioral Variations and Undescribed Behaviors

During video analysis, modifications to previously described behaviors were observed. The protective posture *Retroflex* [21,22], characterized by upturned arms, showed an alteration in this individual where bifurcated arms were crossed to form an X, termed *Retroflex X* (Figure S4). Additionally, adjustments were noted in the feeding behavior of *Web-Overs* [23], also referred to as *Envelope* [24]. Due to the lack of intrabronchial webbing development between bifurcated arms, creating a gap in *Web-Overs*, the studied individual adapted *Web-Overs* tactics. This involved tucking bifurcated arms underneath the body and the crossing of arms L1 and R2 over the bifurcated region or the repositioning of bifurcated arms for optimal coverage (Figure S5; Video S3). Notably, these adjustments were largely employed after a large fish escaped through the gap created by the shorter bifurcated arms during a non-adjusted *Web-Over* (Video S4).

## 4. Discussion

Although arm bifurcation has been morphologically described, to the author’s knowledge, this study presents the best-recorded case of any living cephalopod species with bifurcated arms and the only case wherein the functionality of the bifurcated arms is described. Using observations from an *Octopus* in the wild, we have shown that the usage of bifurcated cephalopod arms partially follows patterns observed in normal arms while also displaying slightly specialized usage hypothesized to be related to their smaller initial size. Further, there existed differentiated usage of the two bifurcated arms, with R1a being used more in *Feeding* behaviors and R1b being used more in *Exploratory* behaviors. Finally, the severity of arm injury directly correlated with usage in *Risky* behaviors, with more severely injured arms being used less in *Risky* behaviors. These results might allude to a degree of pain-associated memory, and that bifurcation may potentially lead to branchial neural differentiation in the affected arms.

### 4.1. Patterns of Arm Use

Despite being morphologically and structurally similar, patterns of association between octopus behaviors and certain arms have been previously described [22,25,26]. Authors have noted specialized functionality within stereotypic motor programs [27,28,29,30], which constitute the majority of the actions made by the seemingly equipotential eight arms. The primary research question aimed to assess the impact of a bifurcated arm on behavioral associations amongst arms. All patterns of usage, besides *Risky* and *Safe* usage, showed predominant differences when examined on a posterior–anterior axis. In the context of locomotion, the individual exhibited a consistent preference for posterior arms, aligning with prior studies [21,22,31,32,33]. However, in *Foraging*, *Feeding*, and *Exploration*, there was no clear distinction between anterior and posterior arms, despite increased anterior arm usage, possibly suggesting a task division of arms not based on an anterior and posterior axis. Further analyses yielded contrasting results, necessitating additional tests for confirmation. This variability in anterior and posterior arm usage may be attributed to the heightened use of R3 in exploratory reach and increased usage distribution on the right side. Contrary to previous findings [14,30,34], anterior arms like R1a and R1b were initially not associated with reaches but were utilized for actions closer to or underneath the body (Video S5). Instead, R3 compensated for the smaller bifurcated arms, particularly in *Foraging* and *Exploratory* behaviors, displayed by its increased initial usage. Over time, as R1a and R1b grew, their usage increased in *Exploration* and *Foraging* behaviors, while R3’s usage within *Exploration* decreased (Video S6). The specialized nature of the hectocotylized R3 arm, in conjunction with these findings, may suggest that food accumulation outweighed the potential reproductive hindrance associated with the loss of R3. These findings differ from the results observed by earlier studies on arm loss in *Octopus*, where male octopuses often hold their R3 arms closer to their body and use them less in *Risky* actions [35,36]. Thus, there might exist anatomical and behavioral distinctions in younger octopuses like the one studied, prioritizing growth over mating.

From the analysis of behavior and arm association conducted in this study, we can corroborate previous findings that octopuses, even those with abnormal limb assemblages, simplify the usage of arms by associating them with behavioral categories [22,25,30]. Specifically, this individual demonstrated a slight favor of the anterior arms when involved in tasks related to foraging or reaching and posterior arms for actions involving benthic locomotion. A behavioral confirmation is noted as being important in laboratory studies due to a lack of environmental relevance [30]. These patterns of association provide additional evidence of the slight specialization that exists within seemingly equipotential arms. Furthermore, the choice of specialization indeed seems to be able to change to best fit the needs of the individual throughout its life, indicating a heightened degree of individualistic behavioral flexibility. Here, the subjects’ decreased usage of bifurcated arms in *Under Web actions*, decreased use of R3 and left-sided arms in *Risky actions*, and increased usage of bifurcated arms in *Risky actions* over time exhibit this personalization.

In eight-armed octopuses, the mirroring of limb specialization on a bilaterally symmetrical axis has been shown, with the R2 and L2 arms being dominant for visually evoked prey capture [25] and R4 and L4 being preferentially used during *Locomotion* behaviors such as *Bipedal Walking* [32,37,38]. As two R1 arms are present in this individual, normal assumptions of arm relatedness were examined. It was hypothesized that R1a and L1 would be the most functionally similar due to their positioning on the body. However, R1b was shown to be more closely related in its usage to L1 rather than R1a. R1a exhibited lower usage in *Exploratory Reach* compared to other anterior arms and exhibited well in its nonuse during an interaction with a hermit crab with three anemones on its shell—note the curling of R1a as all adjacent arms reach out (Video S7). Instead, R1a demonstrated specialization in *Feeding* behaviors, with increased utilization beneath the body and webbing. This suggests that while R1b may function as a typical arm in an eight-armed *Octopus*, the unique anatomy of this individual led to R1a being utilized as a specialized tool.

The continual investigation of specific axial nerve cord transmission pathways has highlighted the bidirectionality of the *Octopus* nervous system, with the bulk of motor control responsibility embedded within the neural circuitry of the arms [28,29,39,40]. Given this, the presence of an extra appendage could surely alter the signals being communicated between the central and peripheral sensory systems. Further, as the brain does not support somatotopic representation, to achieve arm-specific actions, local proprioceptive and tactile input from the periphery is integrated with specific central commands to activate embedded motor programs within the arm site labeled by the initial peripheral input [41,42]. Thus, the distinctive usage of R1a and R1b could stem from the differentiation of the branchial nerve or elsewhere along the axial nerve cord as specific central commands would have been integrated within two unique peripheral sites, generating individual motor programs and, thus, different behaviors.

### 4.2. Persistence of Memory

**Figure 4:**
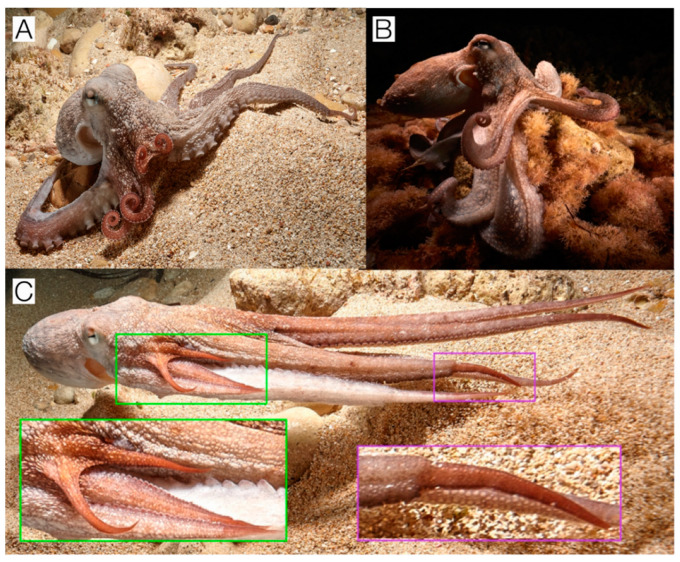
Most common posture displayed by the octopus throughout all the videos; note the curling of the R1a and R1b arms, resembling a curled mustache. (**A**) was taken on 7 December 2021, while (**B**) was taken on 19 April 2022. (**C**) was taken on 7 December 2021, wherein the regrowth of the tip of arm L1 and the regrowth of the smaller bifurcated arms and arms R2 and R3, respectively, are shown in purple and green boxes.

The significant association of arms R1a, R1b, R2, and R3 in *Safe* behaviors, best exemplified through the main posture taken by the R1 arms (Figure 4A,B), and their decreased usage in *Risky* behaviors compared to undamaged arms, may suggest the persistence of a potential trauma or pain-related memory. Post-traumatic protection and avoidance learning have already been observed in cephalopods and, specifically, *Octopus* [43,44,45].

Specifically, it has been demonstrated that this association can result from visual cues, where octopuses avoided locations linked to noxious stimuli. Further, octopuses exhibited wound-directed grooming behaviors at injury sites, indicating the central nervous system’s processing of both injury location and pain quality [44]. In the study subjects’ arm injuries (Figure 4C), with severity of injury following the pattern of R1 > R2 > R3 > L1, avoidance of usage within *Risky* behaviors and usage within *Safe* behaviors aligned with this injury severity pattern, possibly suggesting a memory-driven usage response. This is further implied by the darkened coloration that arms R1–R3 assumed during various foraging outings, a display associated with precursing attacks on conspecifics, predators, and prey [46]. As the display is thought to draw attention to the darkened skin, within this context, it could be interpreted as either a warning or show of aggression to predators or alternatively as a lure to attract prey. Despite its interpretation, as chromatophores, which are responsible for this red coloration, are controlled by a set of lobes in the brain [47], the active darkening of the arms could imply the individual’s understanding, either visually or cognitively, of the previous site of injury. While the injury’s exact cause is unknown, interactions with predators during foraging, particularly affecting those used commonly in *Foraging* and *Feeding* behaviors, often result in the loss of these looser arms [14], and thus may explain the decreased usage of the R1 arms in *Foraging* and their increased use in *Safe* behaviors. Alternatively, previous studies have pointed to the role of visual input in guiding *Octopus* arm actions during exploration, using an angled approach to improve their visual acuity [25,48,49]. Thus, it is also possible that the decreased usage of R1 in foraging could be due to a left-sided preference during approaches, resulting in increased usage of anterior left-sided arms, as was the case in this study. However, further analysis of this individual’s approach angle and neighboring arm recruitment would be necessary to confirm this.

### 4.3. Ecological and Neurological Significance

Efficient and sustainable management of a species can only occur if there is a thorough understanding of the species itself. As cephalopods are short-lived but fast-growing and have a high degree of life-history plasticity (including the ability to alter their RNA to accommodate temperature changes), they have a unique ability to quickly adapt to environmental changes [50,51]. This adaptability has helped cause the proliferation of cephalopod populations despite the decline of many other marine species [52]. Therefore, studying this proliferation through ecology, life history, and behavioral flexibility, as we have done, might provide a unique opportunity to gain insights into conservation not only for cephalopods themselves but for all marine species. Additionally, recent in situ telemetry studies have shown that individuals utilize the same area for extended periods, allowing specific individuals to be studied over longer periods of time [53]. The coupling of these acoustic methods with recent developments in brain recording methods in *Octopus* could help understand individual-specific behaviors and the neurological and mechanistic basis of adaptive arm control [54]. Finally, as members of the genus *Octopus* provide a valuable system for studying regeneration and adaptive control of each of its arms, a deeper understanding of their underlying neurophysiological mechanisms can facilitate significant discoveries and bio-engendering strategies that can be applied to a wide range of taxa (including higher vertebrates) as well as other scientific disciplines, such as regenerative medicine and robotics.

### 4.4. Future Lines of Research

While we have begun to explore bifurcated arm use in *O. vulgaris*, many questions remain. One avenue of research could investigate what happens to neurons in a lost octopus arm—whether they are redistributed throughout the body or simply lost. If neurons are lost, do they slowly regrow, and does the presence of an additional arm result in double the usual number of regrown neurons? Alternatively, if neurons are redistributed throughout the body and then distributed back into the arms as they grow, when an arm is bifurcated, is it possible that the neurons are only distributed into one arm and not the other? If so, this redistribution could potentially explain why bifurcation normally results in a non-functional limb.

Although further research is required to answer these questions, they suggest intriguing possibilities. Could an octopus with newly bifurcated arms be managing with less innervation and effectively learning to use these limbs anew? Or are these limbs fully innervated but simply inhibited by their length? Although these curiosities could prove to be very interesting as future lines of research, at present, they will remain just that, curiosities.

## 5. Conclusions

Although arm bifurcation has been morphologically described, in this study, we have provided an in-depth analysis of arm usage within a unique individual *O. vulgaris* and the only case wherein the functionality of the bifurcated arms is described. This study supports previous research by showing that octopuses, even those with atypical limb morphologies, simplify arm use by associating them with behavioral categories while also displaying specialized usage. Posterior arms were primarily used for locomotion behaviors, while *Foraging*, *Feeding*, and *Exploration* involved both anterior and posterior arms, with initial usage of the hectocotylized R3 arm likely compensating for the smaller bifurcated arms. The bifurcated R1 arms demonstrated specialized usage in tasks close to or beneath the body. Over time, differentiated usage was noted between the bifurcated arms, particularly in R1b’s increased usage in *Exploratory Reach* and *Probe*, while R1a continued to be utilized in *Under Web* actions. This suggests a potential underlying neural differentiation along the branchial or axial nerve cord, where distinct motor programs may have emerged through separate peripheral integration sites. Finally, bifurcated and regrown arms were used more in *Safe* than *Risky* behaviors, with more severely injured arms favoring safer usage. This pattern may indicate an experience-driven adjustment in motor output, aligning with previous observations of avoidance learning and wound-directed behaviors in *Octopus*. However, further investigation is necessary to determine whether these patterns are linked to memory or sensory processing, as they may alternatively reflect a lateralized preference in approach angle.

While this study offers a nearly comprehensive overview of this exceptional individual, it is important to acknowledge that the study was conducted on a solitary subject, lacking replication across multiple individuals. However, the extreme scarcity of live individuals recorded with bifurcated appendages creates a condition where this is not currently possible. This study underscores the behavioral flexibility and adaptive motor control of octopuses, providing deeper insight into the functionality of abnormal regeneration. Future research in this area could offer valuable contributions to marine conservation, regenerative medicine, robotics, and bioengineering by advancing our understanding of neural plasticity and adaptive movement strategies across biological systems.

## Figures and Tables

**Table 1:** Video analysis information. Video numbers 13 and 24 were removed from the study analysis, given their lack of usable segments, and thus were not included in the calculation of total or mean. Totals are indicated in bold. Means are italicized.

Video Number	Recording Date (dd-mm-yy)	Day Correlation (d)	Video Duration (h:min:s)	Percent Analyzed (%)
1	13-12-21	1	0:16:18	96.9
2	12-01-22	31	0:19:51	90.9
3	26-01-22	45	0:5:42	91.1
4	28-01-22	47	0:32:47	98.0
5	30-01-22	49	0:3:48	97.4
6	03-02-22	53	0:23:44	76.0
7	05-02-22	55	0:28:24	95.7
8	06-02-22	56	0:44:36	98.8
9	07-02-22	57	0:32:39	95.1
10	20-02-22	70	0:28:25	89.1
11	21-02-22	71	0:33:27	76.6
12	22-02-22	72	0:41:54	79.9
14	27-02-22	77	0:4:26	88.1
15	03-03-22	81	0:4:31	86.4
16	05-03-22	83	0:40:34	93.8
17	08-03-22	86	0:42:43	96.5
18	10-03-22	88	0:42:06	38.1
19	25-03-22	103	0:25:41	96.2
20	28-03-22	106	0:30:22	77.5
21	10-04-22	119	0:44:46	98.8
22	16-04-22	125	0:41:51	93.4
23	19-04-22	128	0:39:36	91.4
**Total**	-	**128**	**10.46:85**	**-**
Mean	*-*	*72.8*	*0:28:33*	*88.3*

## Data Availability

The original contributions presented in this study are included in the article/Supplementary Material. Further inquiries can be directed to the corresponding authors.

## Supplementary Materials

The following supporting information can be downloaded at https://www.mdpi.com/article/10.3390/ani15071034/s1, Figure S1: Duration and occurrence of arm use for all events; Figure S2: Arm occurrences for all events within behavioral events noted as *Safe* and *Under Web* actions; Figure S3: Raw data plots of *Safe* behavior usage over time for each arm; Figure S4: Normal *Retroflex* behavior displayed by an *Octopus vulgaris*; Figure S5: Alternative *Webover*; Table S1: Behaviors and their different groupings; Table S2: Ethogram of all behaviors, including ones not used in the final analysis; Video S1: example of a non-usable video; Video S2: example of a usable video; Video S3: alteration to *Webover* behavior; Video S4: large fish escapes through the gap created by the smaller bifurcated arms; Video S5: individuals first foraging outing captured on film; Video S6: later foraging outing wherein the bifurcated arms are used more in exploration and other *Risky* behaviors; Video S7: interaction with a hermit crab [55,56,57,58,59,60,61,62].
