# Supplementary material for: The Persistence of Memory: Behavioral Analysis and Arm Usage of a Nine-Armed Octopus vulgaris
Source: Animals (Basel). 2025 Apr 3;15(7):1034. doi: 10.3390/ani15071034 (PMC11987900; doi:10.3390/ani15071034)
Supplement: Supplementary file 1 [file animals-15-01034-s001.zip › animals-3513737-SM.pdf]

SUPPLEMENTARY MATERIAL

Extended Materials and Methods

Photo and Video Specifics

Timeline

A total of 24 videos, spanning 147 days, and 5,933 photos were used in this specific study. The first photo of the octopus was captured on November 8th, 2021 and the final photo was captured on May 10th, 2022, a time period spanning 6 months and 2 days. The videos span from December 13th, 2021 to May 8th, 2022, a time period of 4 months and 25 days. The recording dates were distributed evenly throughout this time period, with 1 video taking place on December, 4, on January, 9, on February, 6, on March, 3, in April, and 1 in May (Table S1). All the data used in the current study were collected in Ibiza, Spain (39°00’36” N 1°17’54” E).

Technical Camera Equipment

Photos were taken using two different Canon cameras, the EOS 1DSMKIII and the EOS 5DMKII. Both models were housed in Subal Housing. The Inon Z-240 and Z-330 strobes were additionally used. Four different lenses were used in the acquisition of photos, these being the the Canon EF 14mm 1:2.8 L II, the Canon EF 24mm 1:1.4 L, the Canon EF 100mm 1:2.8 USM Macro, and finally a Samyang 12mm 1:2.8 AS Fish Eye. All videos were taken using a GOPRO 10, with the AOI-03 Wide Angle Dome lens attachment. Specific details of photo and video settings can be provided by the author upon request.

Method details

Catalog of Behaviors

To understand the focal subject's behavior and identify any deviations from known patterns, a behavior catalog was created using anatomical terminology and existing catalogs for *O. vulgaris* and related species. The catalog, developed in Microsoft Excel (version 16.70), based on 24 videos and supplemented with photos, documented the study subject's behavioral sequences (Table S4).

Behaviors were classified following the widely adopted hierarchical system developed by Packard and his colleagues [55-57]. Packard's hierarchy, consisting of levels based on morphological structures, was adapted for this study, focusing on observable units such as arms, mantle, head, funnel, papillae, and suckers. Components, comprising groups of units, were categorized as locomotor, postural, chromatic, or textural outputs.

The next level in the hierarchy was the overall body pattern, representing the combination of various components. Despite challenges in terminology, body patterns offer insights into the final level—observed behaviors or behavioral sequences. The catalog classified behaviors into categories such as *Locomotion*, *Foraging*, *Feeding*, *Postures*, *Skin Patterns*, *Chromatic Displays*, *Exploration*, *Defense*, *Arm Actions*, and *Other* (Table S1). The distinction between components and observed behavioral sequences was noted in the category section.

SUPPLEMENTARY FIGURES AND TABLES

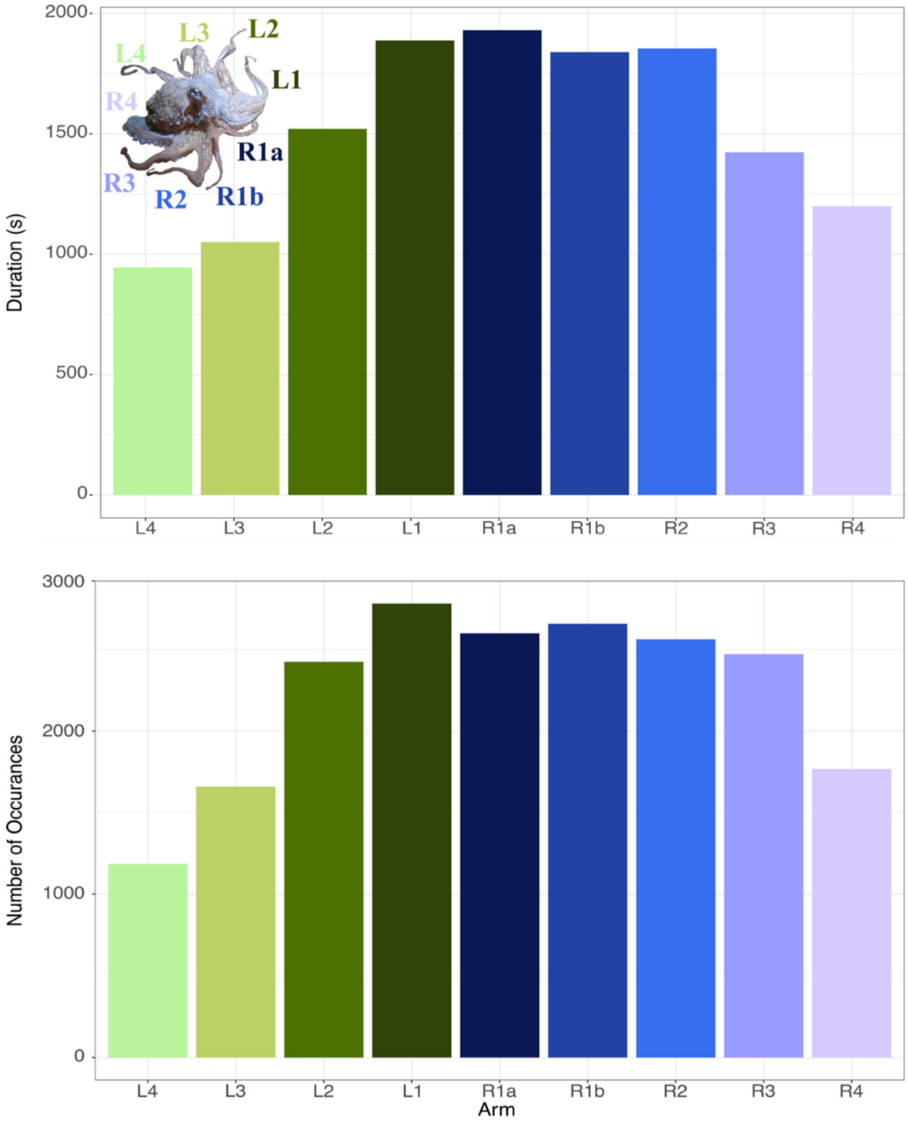

Figure S1.
Duration (above) and occurrence (below) of arm use for all events (n=6442). Photo taken on Jan. 28, 2022 shows terminology of arms.

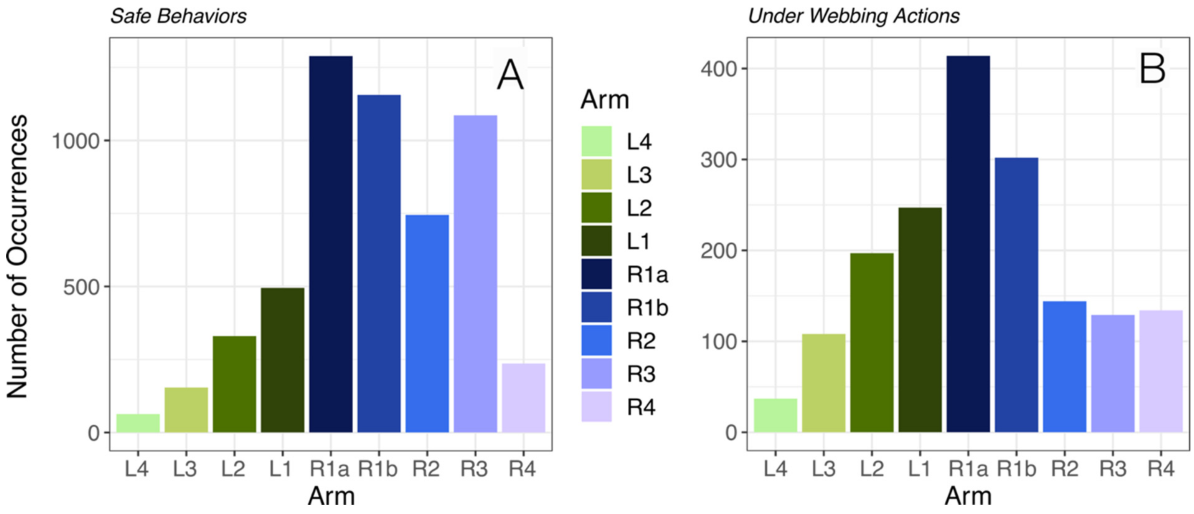

Figure S2.

Arm use occurrences for all events within behavioral events noted as (A) safe and (B) under webbing actions.

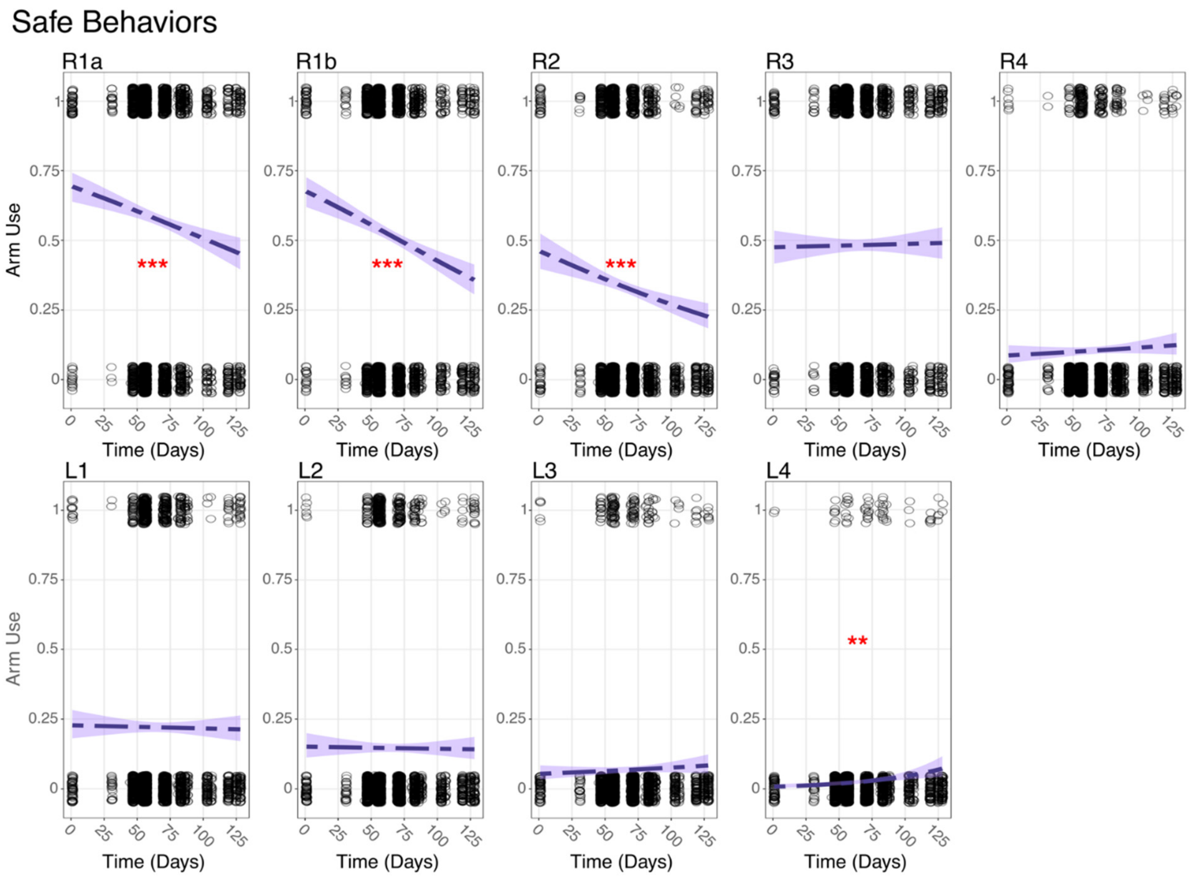

Figure S3.

Raw data plots of safe behavior usage over time for each arm. The logistic regression models were fitted to assess the change in behavior usage over time. The points represent observed data, and shaded areas represent the confidence intervals of the fitted models. The significance level is represented by an asterisk as follows: ** P<0.001, ***P< 0.0001.

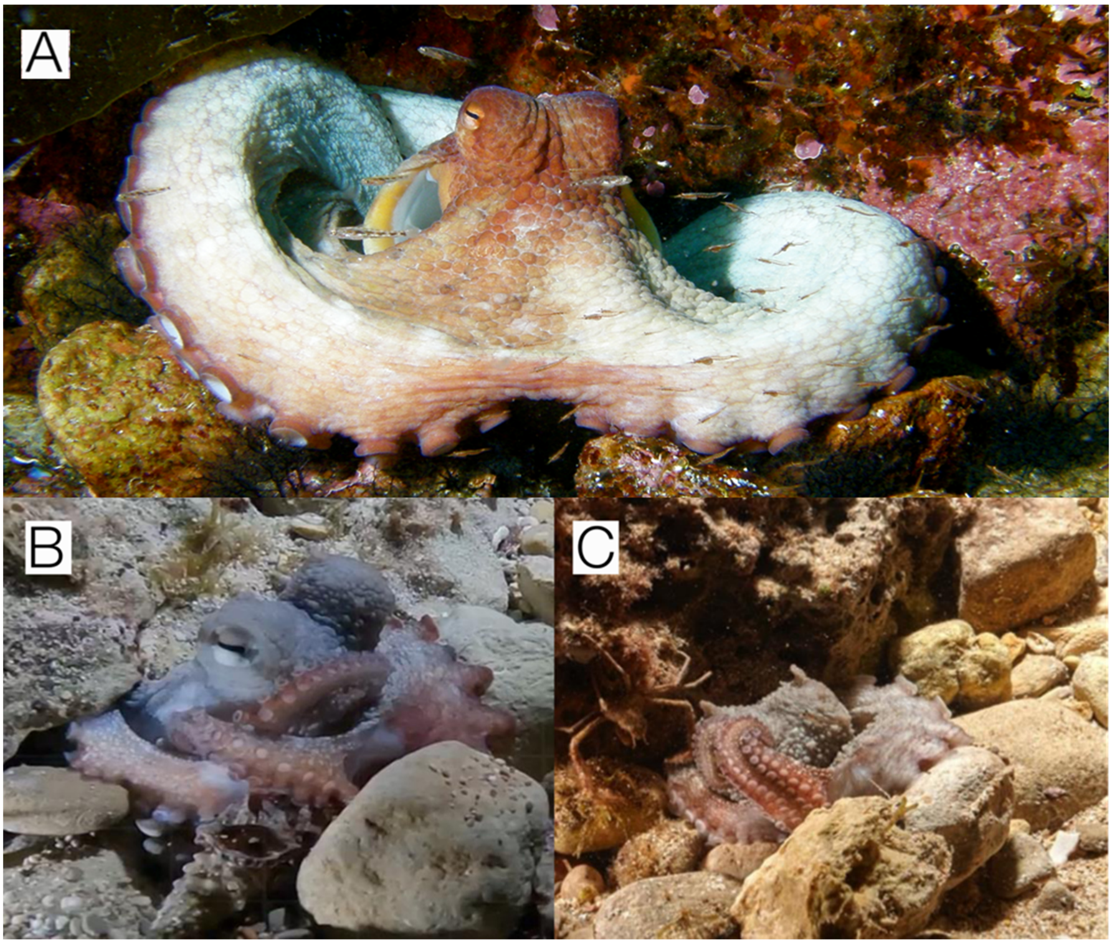

Figure S4.

(A) Normal retroflex behavior displayed by an *Octopus vulgaris*. (B, C) Retroflex X behaviors displayed by the octopus in this study. Note the crossing and raising of the bifurcated arms.

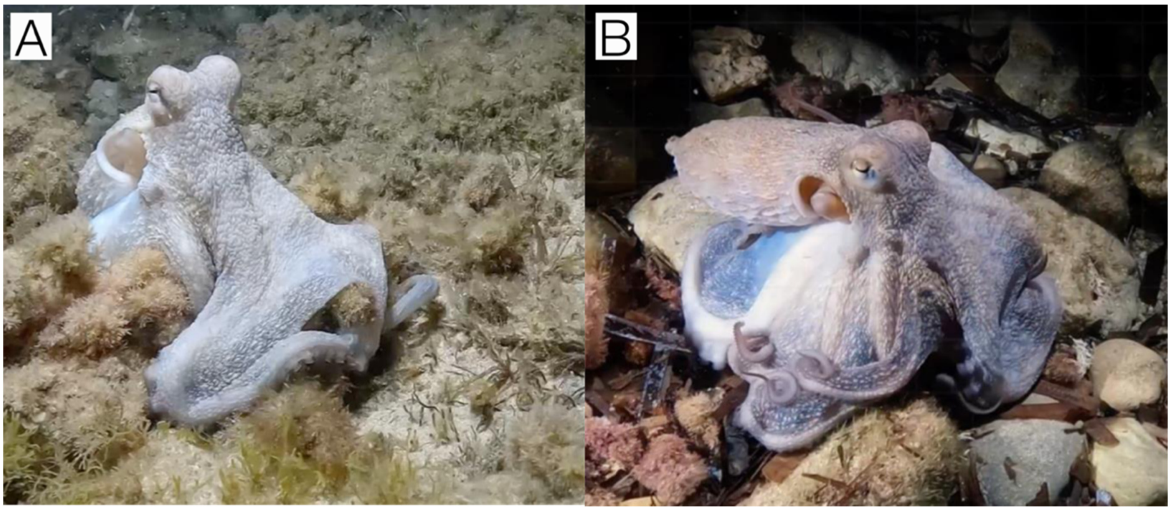

Figure S5.

(A) Photo taken on February 3, 2022, showing crossed alternative Webover wherein L1 and R2 are crossed over each other to close the gap left by the smaller bifurcated arms. (B) Photo taken on May 3, 2022, showing the seatbelt alternative Webover wherein R1b is pulled backward to close the gap caused by the smaller bifurcated arms.

Table S1.

Behaviors and their different groupings. Components indicated with an * designate behaviors that were removed during the two filtration processes and not included in the study. Behavior categories in bold designate those used to test scientific questions.

BEHAVIOR CATEGORY	BEHAVIORS
Alert	Standing Posture
Arm Action	Arm(s)—Pick-up, Arm(s)—Carry, Arm(s)—Push/Pull*
Body Patterning	Passing Cloud Display, Half Passing Cloud Display, Mottle Display*, Mottle Display 1*, Mottle Display 2*
Chromatic*	Unilateral Lightness/Darkness*, Darkened Arm(s)*, Lightened Arm(s)*, Uniform Darkness*, Uniform Lightness*
Cleaning	Cleaning Maneuver
Defense	Inking*, Retroflex
**Explore**	Exploratory Reach, Conflict Posture
**Feeding**	Manipulate, Parachuting Prey, Catch Prey, Prey Lost
**Foraging**	Approach, Pouncing, Webover, Adjusted Webover, Search Webover, Probe
**Locomotion**	Walk, Crawl, Jet*, Free Falling*, Swim*, Tiptoe*
Postural - Arm	Arm(s)—Curved forearm, Arm(s)—Raised, Arm(s)—Curled, Arm(s)—Loose, Arm(s)—Spread (Interbranchial Web Spread), Arm(s)—Tucked Under, Arm(s)—Upturned
**Safe Behaviors**	Arm(s)—Curled, Arm(s)—Curved Forearm, Curled Arm Jet, Conflict Posture, Arm(s)—Tucked Under
**Risky Behaviors**	Exploratory Reach, Crawl, Bipedal Walk, Search Webover, Manipulate, Probe, Catch Prey, Arm(s)—Loose, Arm(s)—Carry, Arm(s)—Pick-Up
**Under Web Actions**	Manipulate, Search Webover, Arm(s)—Tucked Under

Table S2.

Ethogram of all behaviors, including ones not used in the final analysis. The names of the behaviors and their descriptions are included. Additionally, references to previous mentions and/or the alternative names of behaviors are included and cited.

BEHAVIOR	DESCRIPTIONS	REFERENCES
Unilateral Lightness/Darkness	The individual displays uniform coloration on one half of the body, while the other half is often a contrasting color.	Unilateral Effect [57]
Uniform Darkness	The individual displays uniform darkened coloration, often taking the shade of dark red with tints of brown and blue.	Uniform Reddish-Brown [58]
Uniform Lightness	The individual displays uniform lightened coloration, often taking a sand or light grey color with tints of green.	Uniform Light Gray [58]; Chronic Pale [56]
Passing Cloud Display	Uniform dark flush (lasting less than a second) that passes outwards from the head over the dorsal region of the arms and web, often directed towards an external object. Can also be unilateral.	Passing Cloud Display [57]
Half Passing Cloud Display	Uniform dark flush (lasting less than a second) that passes outwards from the head over the dorsal region of the arms and web on one side of the individual, often directed towards an external object.	Half Passing Cloud Display [57]
Mottle Display	Small to moderately sized light and dark splotches of moderate contrast, usually seen over rocks or while an individual is stationary, often the papillae are raised on the mantle and head.	Mottled Phases, Ground Light Grayish-Brown[58]; Chronic General Mottle [58]
Mottle Display 1	Alternative 1 to mottle	Mottled Phases [58]
Mottle Display 2	Alternative 2 to mottle	Mottled Phases [58]
Mottle Display 3	Alternative 3 to mottle	Mottled Phases [58]
Mottle Display 4	Alternative 4 to mottle	Mottled Phases[58]; Broad Conflict Mottle [58]
Darkened Arm(s)	Arms or suckers become darkened in color, usually red but can take on tints of brown	Darkened Arm(s) [59]
Lightened Arm(s)	Arm(s) are lighted achieving a sand or light grey color with tints of green	Lightened Arm(s) [59]
Cleaning Maneuver	Arms are kept close to the body, curled, and twirled repeatedly so that mid to distal suckers are touching the head, mantle, and other arms.	Cleaning Maneuver [57]
Inking	The individual produces a cloud of ink from its funnel; produced in the ink sac. Often followed by jetting	Inking [57]
Retroflex	Arms are spread, raised dorsally, and bent back towards the posterior of the individual so that the suckers are displayed. Often displayed when an individual is at the entrance of a den.	Retroflex [22]
Standing Posture	The individual is stationary, using the mid arms to hold itself in a slightly elevated position. Arms are usually splayed or loosely organized around the individual.	Standing [57]

Conflict Posture	One or more arms (usually one) are extended towards an object while the posterior arms are holding on to an object behind.	Conflict Posture[57]; Cautious Approach [60]4/3/2025 3:24:00 PM
Approach	Normally a precursor to pouncing. The arms are darkened, curled, and sometimes upturned or raised in preparation for a pounce and webover; also occurs during approaches to camera man.	Attack Posture [46]
Pouncing	Arm propelled inferiorly and normally anteriorly around a target, often a rock or algal head. Also includes when individual is lunging at target with arms curled or loosely bent backward. Intermediate step between approach and webover during foraging activities.	Webover [22]
Webover	One of two types of webover, which follow pouncing during foraging. The individual expands interbranchial webbing to try to cover a target (usually a rock or algal head).	Envelope[24]; Webover [22]
Adjusted Webover	One of two types of webover, which follow pouncing during foraging. The individual expands interbranchial webbing to try to cover a target followed by either the inward movement of the two bifurcated arms under the body or by the "seatbelt" method wherein R1a is tucked under but R1b is extended down and posteriorly from the individual. This also occurs when the individual attempts to cover a gap created by smaller bifurcated arms by crossing the adjacent arms (L1) and (R2).	N/A
Search Webover	The arms are tucked under the spread interbranchial webbing to search for food. This action almost always coincides with the behavior Arm(s) Tucked Under.	N/A
Probe	The arms are extended into crevices or under rocks in search of food; form of speculative hunting; often occurs during crawling.	Probe [60]; Groap [61]
Manipulate	The use of arms in passing or moving food underneath the web once prey has been caught.	Manipulate [22]; Take [24]
Parachuting Prey	Prey is caught under spread interbranchial webbing, the mantle expands and is positioned upward, and the webbing expands; both units take balloon or parachute-like shape, finally arms move underneath webbing to move food into mouth.	Parachute [58]
Catch Prey	Often shown firstly through a sharp short movement (almost as if there was a short spasm) while the individual has its web spread over a target item (rock or algal head).	Catch Prey [59]
Prey Lost	Prey is lost or escapes (usually from under the webbing or a searching arm)	Prey lost [59]
Exploratory Reach	The arm(s) are extended out to explore a substrate or object via chemotactile means. The arms are bent dorsally towards the posterior, with the mid-section of the arm leading, followed by an unfurling of the arm so that the distal tip is furthest away from the body. Almost always occurs with mid to distal tips of arms.	Arms Outstretched [57]; Arm Extended [24]; Explore [22]
Walk	Arm(s) (always posterior, and almost always R4 and L4) are bent inferiorly and proximally so that the suckers are making contact with the sediment, then the mid-section of the arm is moved forward in a sequence similar to bipedal walking in humans.	Walking [56]; Walk [22]

Crawl	The octopus moves across the substrate by pulling on the 'leading' arms and pushing with the 'trailing' ones; may also be aided by brief moments of swimming.	Crawl [57]
Free Falling	An individual pushes itself using all arms off a large rock or shelf, then spreads all arms and falls slowly through the water column.	N/A
Jet	An individual moves rapidly through the water column by the expulsion of water through the funnel. The arms are normally extended and straight	Posterior Jet [62]; Swimming (Backwards), Curled Arm Swimming [56]
Swim/Glide	An individual moves slowly through the water column via umbrella-like movements of the arms or interbranchal webs or via posterior expulsion of water from the funnel.	Anterior Jet [62]
Tiptoe	The octopus moves along the substrate by acquisition, hold, and release of several sucker cups per arm and then deflection and reflection of sucker stalks only along a limited proximal length of the arm tubes.	Tiptoe [58]
Arm(s)—Curved Forearm	The arm(s) are partially extended away from the body and bent or curved dorsally, with no more than one curl at the distal tip to expose the sucker. The same arm action is used during the conflict posture.	Reflected Forearm, Curved Forearms [57]
Arm(s)—Raised	The arm(s) are raised dorsally, often with minimal bending or curling of the arms involved.	Arms Raised [56]
Arm(s)—Curled	Arm(s) are curled into rings/circles (more than 1 curl) can be suspended in the air or resting on the sediment close to body.	Arms Tucked In And Curled [57]
Arm(s)—Loose	Arm(s) are loosely organized around the body, normally slack, and often in combination with standing posture.	Arms Loose [57]
Arm(s)–Spread (Interbranchial Web Spread)	The arm(s) are spread and separated from each other, the interbrancial web is spread. Occurs during webovers or during the acquisition of prey.	Interbrachial Web Spread [57]
Arm(s)—Tucked Under	The arm(s) are tucked directly under the head, and possibly the beak and webbing. Often occurs during the manipulation of prey or during risky interactions.	N/A
Arm(s)—Upturned	The arm(s) are raised dorsally, and the distal tip is curved/bent back exposing the suckers. Used during an approach before the arms are thrown ventrally into a webover.	Arms Upturned [57]Arms Raised [56]
Arm(s)—Pick-Up	The arms are used to lift or pick up an item once it has been grasped.	Pick Up [22]
Arm(s)—Carry	An item is carried by the individual (usually under the interbranchial webbing; however, occasionally, away from the body).	Carry [22]
Arm(s)—Push/Pull	Items are pushed or pulled by the arm(s). Suckers are attached to objects and an arm is either pushed or pulled away or towards the individual; often the mid-section of the arm is used.	Push/Pull [22]

SUPPLEMENTARY VIDEOS

Video S1. Video excerpt taken on January 13th, 2022, showing an example of a non-usable video segment due to a lack of visibility of the arms as the study subject moving through marine debris.

Video S2. Video excerpt taken on March 5th, 2022, showing an example of a usable video wherein the study subject "jumps" from a large rock head back towards the substrate.

Video S3. Video excerpt taken on February 3rd, 2022, showing an alteration to the "Webover" behavior, wherein adjacent arms L1 and R2 are crossed over each other to close the gap left by smaller bifurcated arms as they are tucked underneath the mantle in search of food.

Video S4. Video excerpt taken on February 7th, 2022, wherein a large fish escapes through the gap created by the smaller bifurcated arms during a foraging outing.

Video S5. Video excerpt taken on December 13th, 2021, showing the individual's first foraging outing captured on film. Note the constant curling of the two smaller bifurcated arms, in addition to the use of the hectocotylized R3 arm in searching for food.

Video S6. Video excerpt taken on April 10th, 2022, showcasing a later foraging outing wherein the bifurcated arms are used more in exploration and other "Risky" behaviors. Also, note the new decreased use of the R3 arm in searching for food.

Video S7. Video excerpt taken on March 5th, 2022, displaying an interaction the individual had with a hermit crab with anemones growing on top its shell. Note the non-usage of arm R1a in exploration while all adjacent arms are extended toward the hermit crab.
